# Supplementary material for: One-dimensional semimetal contacts to two-dimensional semiconductors
Source: Nat Commun. 2023 Jan 7;14:111. doi: 10.1038/s41467-022-35760-x (PMC9825564; doi:10.1038/s41467-022-35760-x)
Supplement: Supplementary file 1 — Supplementary Information [file 41467_2022_35760_MOESM1_ESM.pdf]

Supplementary information

One-Dimensional Semimetal Contacts to Two-Dimensional Semiconductors

Xuanzhang Li[1], Yang Wei[1*], Zhijie Wang[2], Ya Kong[3], Yipeng Su[1], Gaotian Lu[1], Zhen Mei[1], Yi Su[1], Guangqi Zhang[1], Jianhua Xiao[1], Liang Liang[1], Jia Li[2], Qunqing Li[1], Jin Zhang[3], Shoushan Fan[1], and Yuegang Zhang[1*]

[1]State Key Laboratory of Low-Dimensional Quantum Physics, Department of Physics and Tsinghua-Foxconn Nanotechnology Research Center, Tsinghua University, Beijing 100084, China

[2]Shenzhen International Graduate School, Tsinghua University, Shenzhen 518055, China

[3]College of Chemistry and Molecular Engineering, Peking University, Beijing 100871, China

*Email: weiyang@tsinghua.edu.cn, yuegang.zhang@tsinghua.edu.cn

Supplementary Notes

Note 1. Desulfurization process

The annealing process performed under vacuum at 300 °C for 1h can achieve complete desulfurization without affecting the transport properties of both SWCNT and MoS_2. At the same time, the clean and tight van der Waals contact can be formed between CNT and MoS_2. The FETs with fresh CNT and MoS_2 channels were fabricated to confirm the desulfurization results experimentally, as shown in Supplementary Fig. 5 and 6. Supplementary Fig. 5a and 6a present the optical images of the devices before sulfur deposition, after sulfur deposition and after annealing. There are no sulfur particles around the devices after annealing treatment. Supplementary Fig. 5b and 6b show the corresponding transfer curves. After sulfur deposition, the transfer characteristics of the CNT FET became gate-independent, and the threshold voltage of the MoS_2 FET shits positively and the on current decreases significantly. While the transfer curves of the CNT and MoS_2 FETs after annealing treatment are almost coincide with those before sulfur deposition. These results indicate that the sulfur particles can be completely removed by the annealing treatment and the annealing procedure carried out under vacuum does not lead to a significant healing of sulfur vacancy in MoS_2.

Note 2. Extraction of Schottky barriers.

The barrier heights at the CNT-MoS_2 and Ti-MoS_2 interfaces can be extracted using the 2D thermionic emission model[1]. The source-drain current through a barrier into the 2D channel can be expressed as:

$$I_d = AT^{3/2}\exp\left(\frac{-q\varphi_B}{k_BT}\right)\left[\exp\left(\frac{qV_d}{nk_BT}\right)-1\right]$$

where I_d, A, T, q, φ_B, k_B, V_d is the drain current, the effective Richardson constant, temperature, electronic charge, the SBH, the Boltzmann constant, and source-drain bias, respectively. n is the nonideal factor of the Schottky diode. The Arrhenius plots, slope of which is $-q\varphi_B + qV_d/n$, are drawn to extract the SBHs corresponding to different gate voltages.

Note 3. Extraction of resistivity of SWCNT

Since there are only two conductive channels near the Fermi level of metallic SWCNT, the total resistance of a long-range SWCNT device can be expressed as[2]:

$$R_{CNT} = \frac{h}{4e^2}\frac{L}{L_{mfp}} + 2R_{nc}^{CNT} + R_Q^{CNT}$$

where the $\frac{h}{4e^2}\frac{L}{L_{mfp}}$ represents the wire resistance of the SWCNT channel, L is the channel length of SWCNT and L_{mfp} is the mean free path of electrons; R_{nc}^{CNT} is the interfacial resistance caused by the imperfect contact between metal and SWCNT, $R_Q = 6.5k\Omega$ is the quantum resistance determined by the number of conductive channels, these two items are collectively referred to as the contact resistance of CNT. According to equation above, we plot the transfer characteristics of the several 2-terminal CNT FETs with different channel lengths in Figure 1c, then extract the contact resistance R_{nc}^{CNT} and channel resistivity $\rho_{CNT}^{on\,SiO_2} = \frac{h}{4e^2}\frac{1}{L_{mfp}}$ corresponding to each V_g through the transfer length method (inset of Supplementary Figure 15a). Note that the resistance induced by the electron scattering at the junctions between CNTs with and without MoS_2 contacting can be neglected due to the linear dispersion relationship of the metallic SWCNT and the mismatched momentum between its two conducting channels[3-5]. Thus, resistivity of the CNT segments contacting with MoS_2 channel ($\rho_{CNT}^{on\,MoS_2}$) can be further calculated by subtracting the contact resistance and wire resistance of the CNT (on SiO_2) from the 2-terminal resistance (electrodes 1 and 9, Supplementary Figure 16c).

Note 4. CIFS and band alignment analysis.

The gate-tunable contact-induced Fermi level shift (CIFS) in semimetal has been discussed in our previous work[6]. It indicates that the Fermi level of the SWCNT electrode in contacting with TMD channel is misaligned with that of the SWCNT wire on SiO_2 substrate due to the Sm-S interface charge transfer (Figure 2e). On the basis of the as-developed Resistivity Comparison Methodology and Fermi Level Catch-up Model, the CIFS in CNT electrodes under different V_g has been illustrated by the band diagrams (Supplementary Figure 15c) corresponding to the 5 regions (A to E) in the comparative resistivity plot (Figure 3b, Supplementary Figure 15b). In Region A, the Fermi level of CNT is lower than that of MoS_2, the P-type CNT receives electron injection and the conductivity decreases. Then from A to B, CNT's Fermi level catches up with and surpass MoS_2 at β. Region B indicates that the P-type CNT accepts hole injection and the conductivity is improved. Then the conductive type switching occurs at Region C and D, where the N-type CNT accepts hole injection and then reverses into a P-type one. Point X indicates where the CNT with and without MoS_2 contacting have different conducting types but the same resistivity, revealing that their Fermi levels are symmetric with respect to the Dirac point. Region E represent that the N-type CNT accepts hole injection, the N-doping is weakened and the conductivity is reduced. On this basis, the Fermi level positions of CNT and MoS_2 before and after contact formation as well as the direction of built-in electric field in MoS_2 after contact formation can be confirmed (Supplementary Figure 15d, 15e). Just as shown in Figure 2g, the built-in electric field points from CNT to MoS_2, and the band of MoS_2 bends upward in junction region when V_g>-13V. The Fermi level of the CNT wire on SiO_2 and the CNT electrode on MoS_2 approaches their Dirac points at V_g=-8V and V_g=37V, respectively. It should be noted that the resistivity change of the CNT electrode on MoS_2 tends to be weak at

V_g=20V~50V, which is due to the enhanced electrostatic screening introduced by the bottom MoS_2. Thus, the gate modulation efficiency of CNT electrode's Fermi level decreases and its Fermi level is confined near the Dirac point when the device is switched to the on state.

Note 5. Gate modulation of the potential barrier at the semimetal-semiconductor (Sm-S) junctions

The band diagrams of Sm-S (n-type) junctions have been sketched in Supplementary Figure 14. The potential barrier at the interface Φ_B can be expressed as:

$$\Phi_B = \begin{cases} SBH, \ \phi_s < 0 \\ \phi_n, \ \ \phi_s > 0 \end{cases}$$

where SBH, ϕ_n, $\phi_s = W_{SC} - W_{Sm}$ are Schottky barrier height, the energy difference between the work function and affinity of the semiconductor, the surface potential of the semiconductor, respectively. For $\phi_s < 0$, $\Phi_B = SBH = W_{Sm} - \chi_{SC}$, where W_{Sm} and χ_{SC} are the work function of semimetal and the affinity of semiconductor respectively. For $\phi_s > 0$, $\Phi_B = \phi_n = SBH + \phi_s = W_{Sm} - \chi_{SC} + \phi_s = W_{SC} - \chi_{SC}$, where W_{SC} is the work function of semiconductor.

When an external electrical field is applied, the work functions of both semimetal and semiconductor are tuned following $\frac{1}{e}\frac{dW}{dV_g} = -\frac{1}{e}\frac{d\mu}{dV_g} = -\frac{C_g}{C_Q+C_{it}+C_g}$, where the quantum capacitance C_Q is proportional to the DOS, C_{it} is the interface trap capacitance caused by the surface states, $C_g$is the gate capacitance of SiO_2 dielectric. At V_g=-13V~50V, the work function of CNT is smaller than that of MoS_2 as shown in Supplementary Figure 15e, which means $\phi_s > 0$ and the potential barrier $\Phi_B = W_{MoS_2} - \chi_{MoS_2}$. In this way, the dependence of the barrier on V_g can be expressed as

$$\frac{1}{e}\frac{d\Phi_B}{dV_g} = \frac{1}{e}\frac{dW_{MoS_2}}{dV_g} = -\frac{C_g}{C_{Q,MoS_2}+C_{it}+C_g}.$$

When the Fermi level of MoS_2 is located in the band gap, the $C_{Q,MoS_2} = 0$ and $\frac{1}{e}\frac{d\Phi_B}{dV_g} = -\frac{C_g}{C_{it}+C_g}$. Therefore, the Φ_B is linearly correlated with V_g and the conduction current in the device increases exponentially (corresponding to the subthreshold region), which is consistent with the low V_g regime (V_g < 10V) in Figure 2a and 2f. When the Fermi level of MoS_2 approaches its conduction band edge or the donor level, the C_{Q,MoS_2} increases and the $\frac{d\Phi_B}{dV_g}$ is not a constant anymore and decreases, as shown in the high V_g regime (V_g > 15V) in Figure 2f. The device switches to the on state.

Note 6. Longitudinal transmission line model.

As the size of the MoS_2 FET with 1D semimetal contacts is in the order of microns, the wire resistance of the CNT electrodes cannot be ignored. Part of the voltage will be distributed on the electrodes when bias voltage applied, so that the current injected from CNT to MoS_2 is uneven in the y direction (Supplementary Figure 16a). Considering the symmetric conditions of our device, the potential drop on the two CNT segments can be assumed to be the same, and the current in MoS_2 is parallel to the channel direction. Taking the two intersections (orange and green points in Figure 3a, Supplementary Figure 16b) of CNT and the edge of MoS_2 as the boundary, the potential conditions can be set as: $V_1(0) = V_0$ and $V_2(0) = 0$. According to the Kirchhoff's law, the differential equations of potential and current distribution on the CNT-1 are:

$$\begin{cases} \frac{dI}{dy} = -\frac{2V - V_0}{R_t} \\ \frac{dV}{dy} = -I\rho_l \end{cases}$$

where $R_t = \frac{2r_c}{D_{CNT}} + \rho_{TMD}^{2D}L$ (Ω·μm) is defined as the resistance to be overcome when current is injected into MoS_2 channel and transport along the *x* direction; $\rho_l = \rho_{CNT}^{on\ MoS_2}$ (Ω·μm^{-1}) is defined as the resistance to be overcome when the current is transmitted in CNT wire along the *y* direction. Therefore,

$$\frac{d^2}{dy^2}V - \frac{2\rho_l}{R_t}V + \frac{\rho_l V_0}{R_t} = 0$$

The solution is:

$$\begin{cases} V_1(y) = \frac{V_0}{2}e^{-\frac{y}{L_T}} + \frac{V_0}{2} \\ I_1(y) = \frac{V_0}{\sqrt{2R_t\rho_l}}e^{-\frac{y}{L_T}} \end{cases}$$

where $0 \leq y \leq W$. The transfer length $L_T = \sqrt{\frac{R_t}{2\rho_l}} = \left[\frac{\frac{2r_c}{D_{CNT}} + \rho_{TMD}^{2D}L}{2\rho_{CNT}^{on\ MoS_2}}\right]^{\frac{1}{2}}$ (μm) is a characteristic length to evaluate the decay rate of current density along the y direction.

Then the transverse current density in the channel is:

$$J_{TMD}(y) = -\frac{dI_1(y)}{dy} = \frac{V_0}{R_t}e^{-\frac{y}{L_T}}\ (\mu A \cdot \mu m^{-1})$$

The total current in channel is:

$$I_{tot} = \int_0^W J_{TMD}dy = \frac{V_0}{R_t}\int_0^W e^{-\frac{y}{L_T}}dy = \frac{V_0 L_T}{R_t}\left(1 - e^{-\frac{W}{L_T}}\right) = \frac{V_0}{\sqrt{2R_t\rho_l}}\left(1 - e^{-\frac{W}{L_T}}\right)$$

Thus the equivalent resistance of the heterostructure can be expressed as:

$$R_H = \frac{\sqrt{2R_t\rho_l}}{1 - e^{-\frac{W}{L_T}}} = \sqrt{2R_t\rho_l}\Bigg/\left(1 - e^{-\frac{W}{\sqrt{\frac{R_t}{2\rho_l}}}}\right)$$

And the total resistance of the MoS_2 FET with 1D contacts is:

$$R_{tot} = (2R_{nc}^{CNT} + R_Q^{CNT}) + 2\rho_{CNT}^{on\ SiO_2}L_{in} + (R_H + R_Q^{CNT})$$

Note 7. Contact resistance in short contact limit.

For general 2D/3D contacts, the contact resistance R_c (in Ω·μm) is determined by the interface contact resistivity r_c (in Ω·cm^2) and the channel sheet resistivity ρ^{2D} (in Ω·□$^{-1}$), which can be expressed by the transmission line model[7,8] as:

$$R_c = \sqrt{\rho^{2D}r_c}coth(\frac{l_c}{l_t})$$

where l_c is the contact length. The current transfer length $l_t = \sqrt{r_c/\rho^{2D}}$ is defined to describe the current crowding effect at the electrode edge. It presents the average distance that an electron (or hole) travels in channel beneath the contact before it enters the contact. (Supplementary Figure 16d) When $l_c \gg l_t$, $R_c = \sqrt{\rho^{2D}r_c}$ is independent of the contact length; while when $l_c \sim l_t$ or $l_c < l_t$,

R_c increases exponentially with the decrease of contact length (curves in Figure 3e). In short contact limit ($l_c \ll l_t$ or $l_c/l_t \to 0$), the equation can be simplified as $R_c = r_c/l_c$.

In the case of the 1D semimetal contact, there is no current crowding phenomenon and the current is directly injected into the channel from the contacting point. Thus, the injection efficiency of electrons from electrode to channel is greatly improved, and the contact resistance at the short contact limit shall be expressed as $R_c = r_c/D_{CNT}$, which is independent of the channel sheet resistivity ρ^{2D}. Furthermore, when the Ohmic contact is formed, the interface contact resistivity r_c caused by electron tunneling through the van der Waals gap determines the contact resistance R_c.

At low bias, the tunnelling-specific resistivity is[9,10]

$$r_t \approx \frac{4\pi^2\hbar w_t^2}{e^2}\frac{exp[2\frac{(2m_e)^{1/2}}{\hbar}\alpha w_t\phi_t^{1/2}]}{\frac{(2m_e)^{1/2}}{\hbar}\alpha w_t\phi_t^{1/2}-1}$$

where w_t, ϕ_t, α, m_e and $\hbar$ is the tunneling (vdW) gap width, the tunneling barrier height, the empirical factor that is associated with the shape of the barrier, the free-electron mass and the reduced Planck's constant respectively. According to the DFT calculation results, for (5,5)SWCNT/MoS_2 heterostructure, w_t=1.87Å and ϕ_t=4.03eV. Therefore, the corresponding $r_t \approx 2.882 \times 10^{-9}\,\Omega\cdot\mathrm{cm}^2$, and the contact resistance $R_c = r_t/D_{CNT} = 419\,\Omega\cdot\mu m$. The $D_{(5,5)CNT} = 0.6875nm$.

Note 8. Calculation of the maximum on-state current density.

On the perspective of FETs, the current density should be expressed as:

$$J = \frac{I_{tot}}{W} = \frac{V_0}{R_t}\frac{1-e^{-\frac{W}{L_T}}}{W} \quad (\mu A\cdot\mu m^{-1})$$

The experimentally measured L_T is about 10μm when the device is switched to the on state (Supplementary Figure 17b). Thus, $1-e^{-\frac{W}{L_T}} \to 0$ when $W < 0.1\,\mu m$.

$$\lim_{W\to 0}\frac{1-e^{-\frac{W}{L_T}}}{W} = \lim_{W\to 0}\frac{\frac{1}{L_T}e^{-\frac{W}{L_T}}}{1} = \frac{1}{L_T}$$

$$\lim_{W\to 0} J = \frac{V_0}{\sqrt{2R_t\rho_l}}\frac{1}{L_T} = \frac{V_0}{R_t} \quad (\mu A\cdot\mu m^{-1})$$

That is, the current distribution in channel is nearly uniform when the channel width *W* is less than 100nm. The theoretical on-state current density of the FETs with 1D semimetal contact can be calculated by substituting the measured r_c (R_t) into the above formula. For r_c=1×10^{-6} Ω·cm^2 (experimental measured), J_{max}=5μA·μm^{-1}.

Note 9. Calculation of the field-effect mobility.

The field-effect mobility of our device can be extracted from the transfer characteristics using the equation $\mu = [dI_{ds}/dV_g] \times [L/WC_{ox}V_d]$, where C_{ox} = 11 nF·cm^{-2} is the back gate capacitance (300 nm SiO_2). The mobility of the CNT-MoS_2 FETs reaches peak values of 14 $cm^2V^{-1}s^{-1}$ and 5.4 $cm^2V^{-1}s^{-1}$ for the 4L and 1L channel, while the Ti-MoS_2 FETs show smaller values of 7.6 $cm^2V^{-1}s^{-1}$ and 0.3 $cm^2V^{-1}s^{-1}$ respectively. However, it should be noted that the mobility of the CNT-MoS_2 FETs calculated in this way is far smaller than the actual situation due to the uneven current distribution

in channel. A modification to the current density $J_{ds} = I_{ds}/W$ is necessary. Considering the maximum current density (5μA·μm^{-1}) we have deduced, the mobility of the 4L and 1L MoS_2 FETs were calculated to be 85.9 cm^{2}V^{-1}s^{-1} and 69.3 cm^{2}V^{-1}s^{-1}.

	Current Density (V_d=1V)	Mobility	Modified Mobility
4L MoS_2 FET (CNT contact)	0.814 μA·μm^{-1}	14 cm^{2}V^{-1}s^{-1}	85.9 cm^{2}V^{-1}s^{-1}
1L MoS_2 FET (CNT contact)	0.375 μA·μm^{-1}	5.4 cm^{2}V^{-1}s^{-1}	69.3 cm^{2}V^{-1}s^{-1}
4L MoS_2 FET (Ti contact)	0.184 μA·μm^{-1}	7.6 cm^{2}V^{-1}s^{-1}	/
1L MoS_2 FET (Ti contact)	0.012 μA·μm^{-1}	0.3 cm^{2}V^{-1}s^{-1}	/

Note 10. Method to estimate the gate-dependent Fermi level position of CNT.

The Fermi level positions (μ) and work functions ($\phi = 0 - \mu$) of low-dimensional materials can be controlled by V_g through electrostatic capacitance effect following $\frac{1}{e}\frac{d\mu}{dV_g} = -\frac{1}{e}\frac{d\phi}{dV_g} = \frac{C_g}{C_Q+C_g}$, where C_g, C_Q is the gate capacitance and quantum capacitance[11], respectively. The acknowledged value of the quantum capacitance of metallic SWCNTs is 0.2fF·μm^{-1} (between the two first von Hove Singularities)[12]. The gate capacitance of CNT transistors with 20-nm thick dielectric layer is $C_g = \frac{2\pi\varepsilon_0\varepsilon_r}{\ln{(2+\frac{2t}{r})}}$, where ε_0 is the permittivity of vacuum, $\varepsilon_r = 3.55$ is the relative dielectric constant of h-BN, $t = 20nm$ is the dielectric thickness and r is the radius of SWCNTs. Specially, $r_{(5,5)}$=0.344nm and $r_{(12,12)}$=0.825nm. We therefore estimate that $C_{g,(5,5)}$ = 0.04135 fF·μm^{-1} and $C_{g,(12,12)}$ = 0.05033 fF·μm^{-1}. Then

$$\left.\frac{d\mu}{dV_g}\right|_{(5,5)} = 0.171, \quad \left.\frac{d\mu}{dV_g}\right|_{(12,12)} = 0.201$$

Supplementary Figures

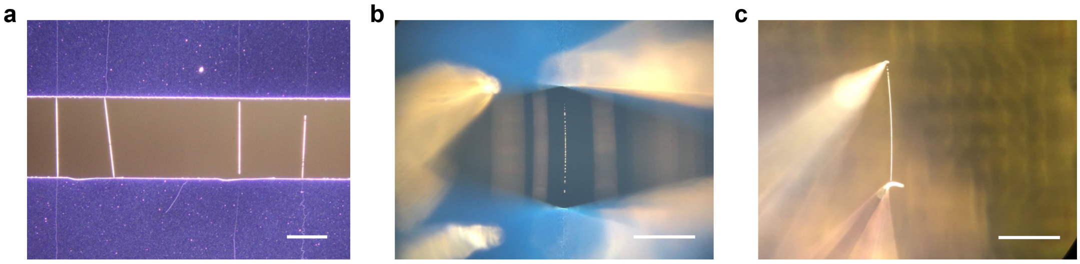

Supplementary Fig. 1 Optical image of the sulfur-treated ultralong CNT. a, Ultralong CNTs on the trenched Si/SiN_x substrate. Scale bar: 100 µm. **b**, CNTs prepared to be transferred using tungsten tips. Scale bar: 100 µm. **c**, CNT transferred from substrate. Scale bar: 100 µm. The condensed morphology of sulfur vapor on CNTs reflects the diameter of CNTs, and the sparser distribution of sulfur droplets always indicates the smaller diameter (**b**).

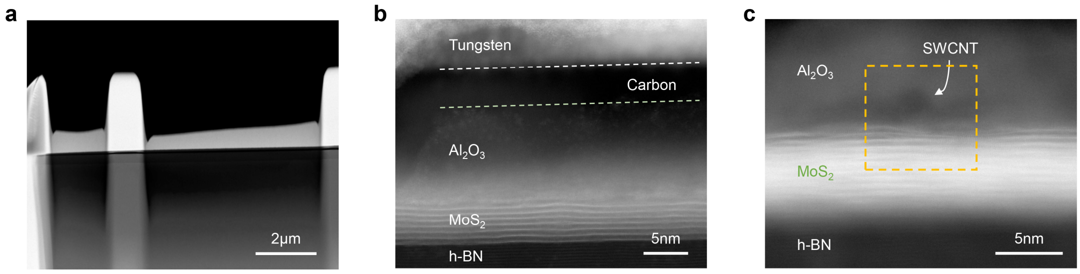

Supplementary Fig. 2 STEM analysis. a, The as-fabricated FIB sample. **b**, Cross-sectional STEM image of the MoS_2 channel. The Al_2O_3 grown by atomic layer deposition is used as the protective layer in FIB process, while the evaporated carbon layer is to enhance the conductivity of the sample surface. The top tungsten is deposited by the electron beam and ion beam successively, which is also used as a protective layer. The h-BN is used as the substrate of the heterostructure. **c**, Cross-sectional STEM image of the CNT-MoS_2 interface. The yellow dotted box presents the mapping area shown in Fig.1b.

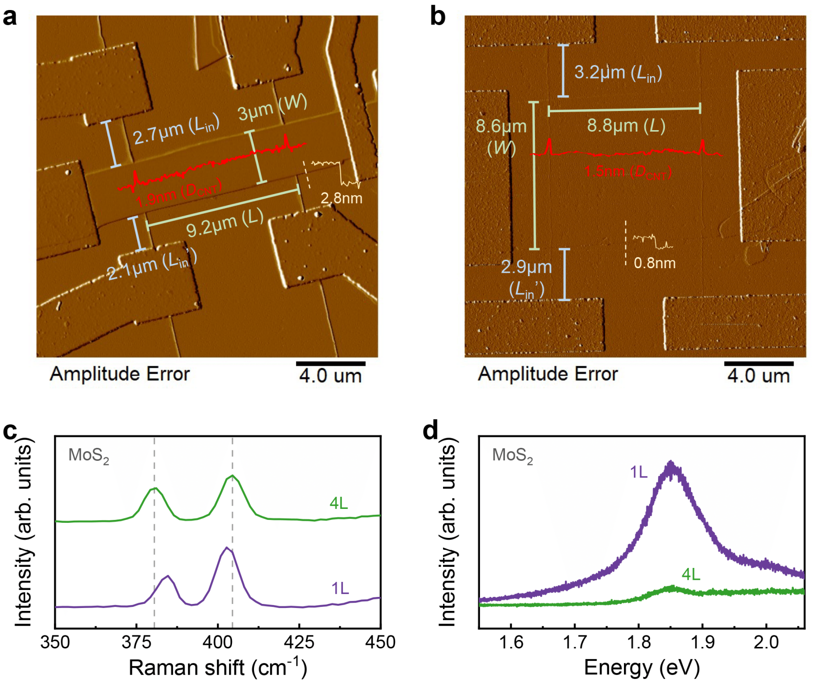

Supplementary Fig. 3 Atomic force microscope, Raman spectra and Photoluminescence spectra of the MoS_2 channels. **a**, AFM peak force error image of the CNT-contact 4L-MoS_2 FET. **b**, AFM peak force error image of the CNT-contact 1L-MoS_2 FET. The interconnect length of CNT wire L_{in}, diameter of CNT D_{CNT}, channel length L, channel width W and channel thickness are all marked. **c**, Raman spectra of the 1L and 4L MoS_2. D, Photoluminescence spectra of the 1L and 4L MoS_2.

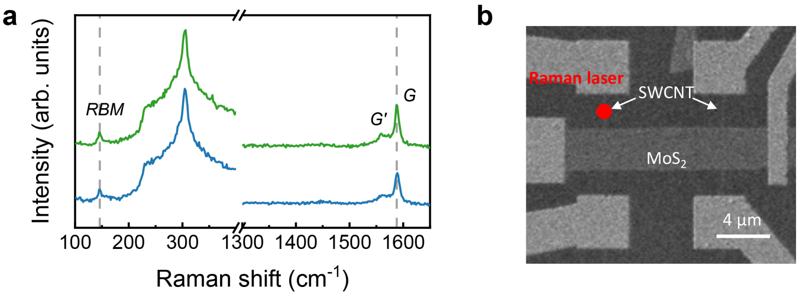

Supplementary Fig. 4 Raman spectra of the SWCNTs. **a**, The raw Raman spectra of the CNTs on Si/SiO_2 substrate in range of 100 cm^{-1} to 400 cm^{-1} to confirm the RBM mode. The 303 cm^{-1} peaks come from the Si/SiO_2 substrate[13]. **b**, The SEM image of the CNT-contacted MoS_2 FET. The Red point indicates the position of the focused Raman laser beam.

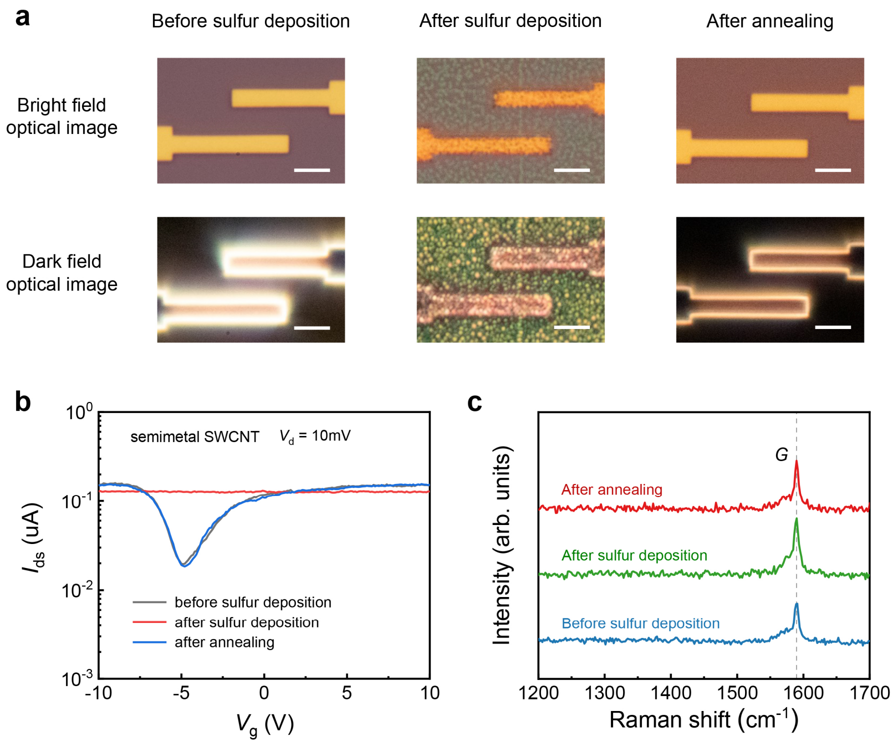

Supplementary Fig. 5 Characterization of the effect of the sulfur deposition and annealing process on CNT. a, Optical images of a Ti-contacted CNT FET with semimetal CNT channel before and after sulfur treatment. Scale bar: 4 μm. **b**, Transfer characteristics of the CNT FET before and after sulfur treatment. **c**, Raman spectra of the CNT before and after sulfur treatment.

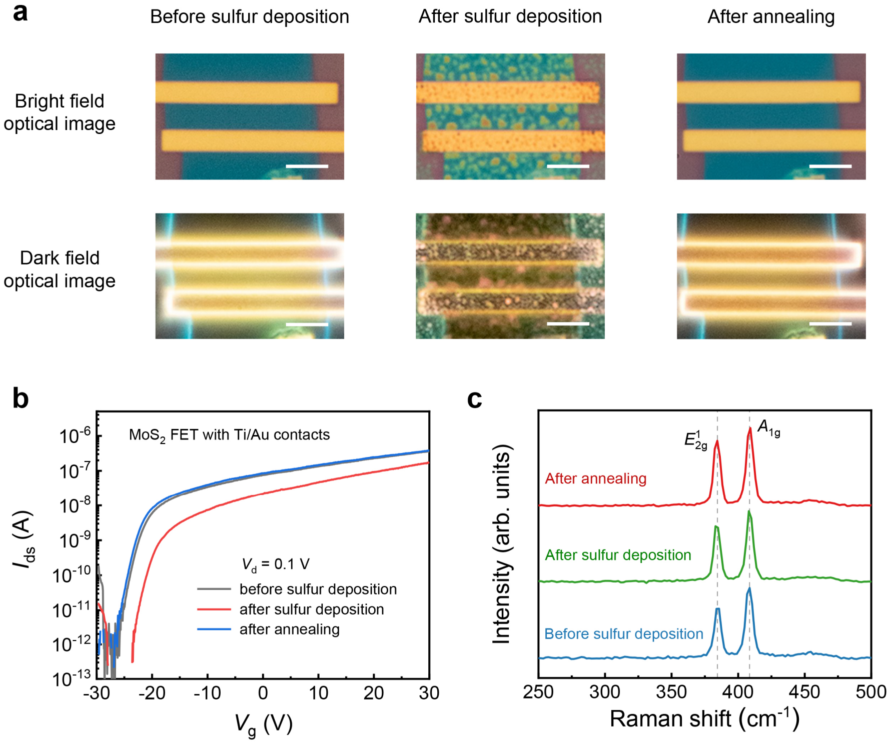

Supplementary Fig. 6 Characterization of the effect of the sulfur deposition and annealing process on MoS_2. a, Optical images of a Ti-contacted MoS_2 FET before and after sulfur treatment. Scale bar: 4 μm. **b**, Transfer characteristics of the MoS_2 FET before and after sulfur treatment. **c**, Raman spectra of the MoS_2 before and after sulfur treatment.

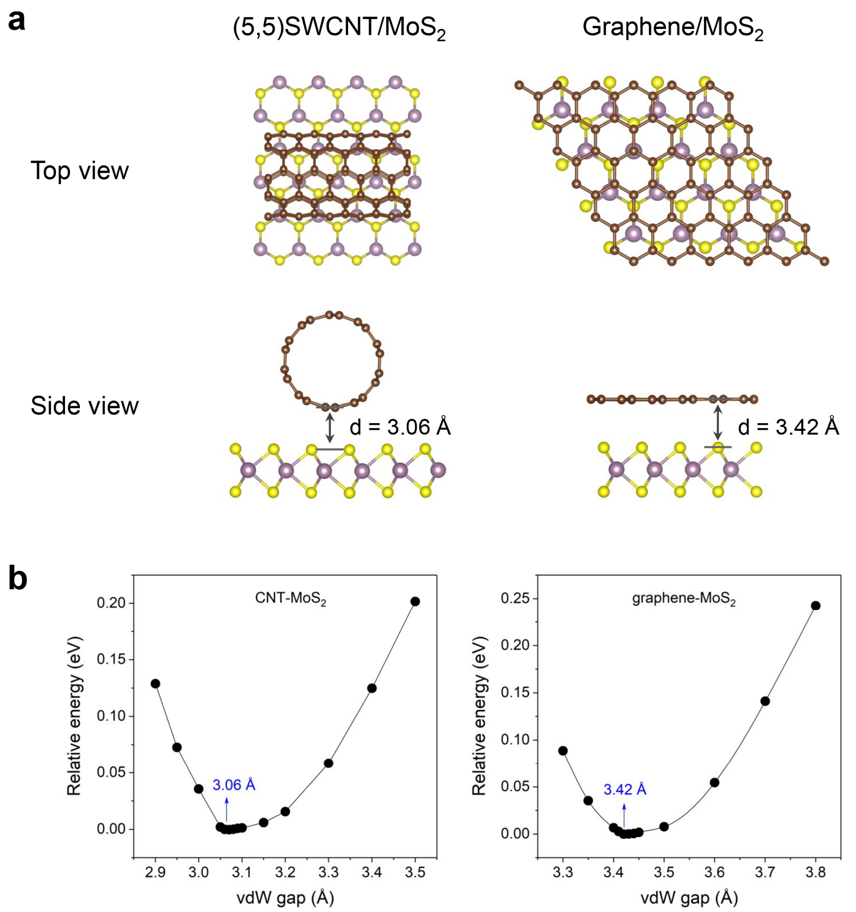

Supplementary Fig. 7 a, Top and side views of the CNT/MoS_2 and graphene/MoS_2 heterostructures. **b**, Relative energy of CNT/MoS_2 and graphene/MoS_2 heterostructures as a function of vdW gap. The CNT/MoS_2 presents obviously smaller vdW gap due to the tubular structure of CNT.

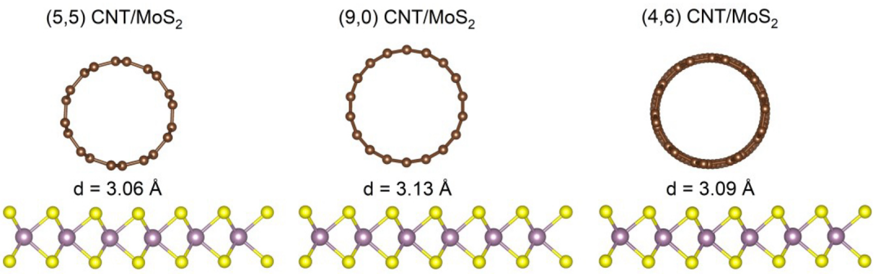

Supplementary Fig. 8 Side views of the as-calculated (5,5) CNT/MoS_2, (9,0) CNT/MoS_2 and (4,6) CNT/MoS_2 heterostructures.

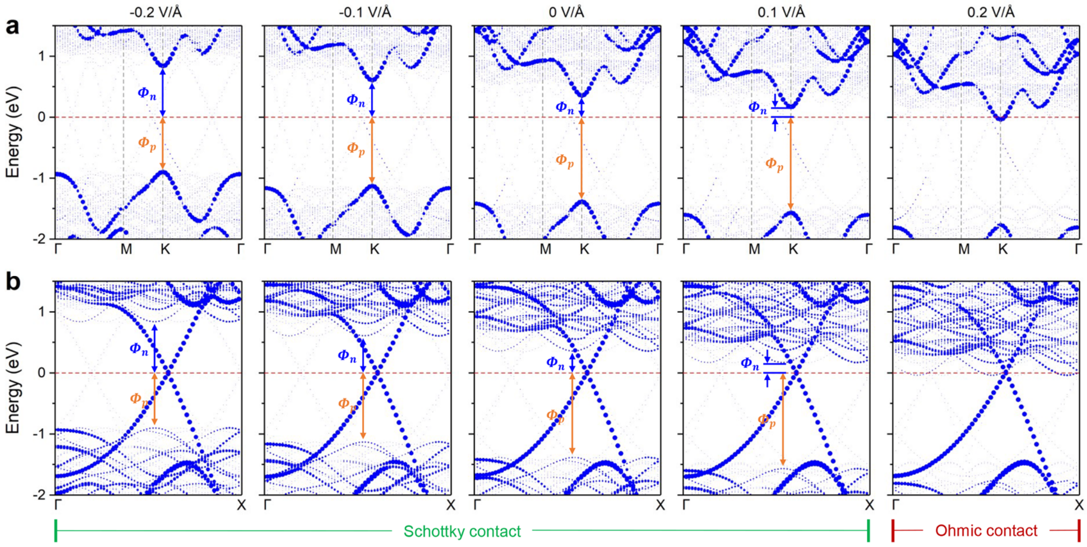

Supplementary Fig. 9 Unfolded band structures of (a) MoS_2 and (b) CNT in the CNT/MoS_2 heterostructure under different external electric fields.

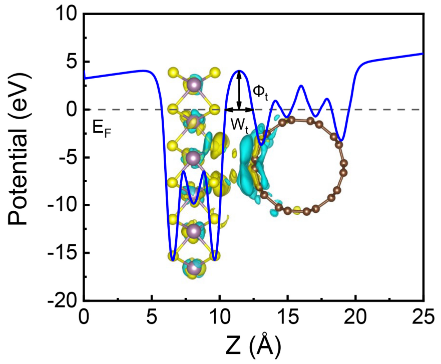

Supplementary Fig. 10 Electrostatic potential of the (5,5) CNT/MoS2 heterostructure under applied electric field of 0.2 V/Å. The potential barrier width w_t and barrier height Φ_t are 1.87 Å and 4.03 eV, respectively.

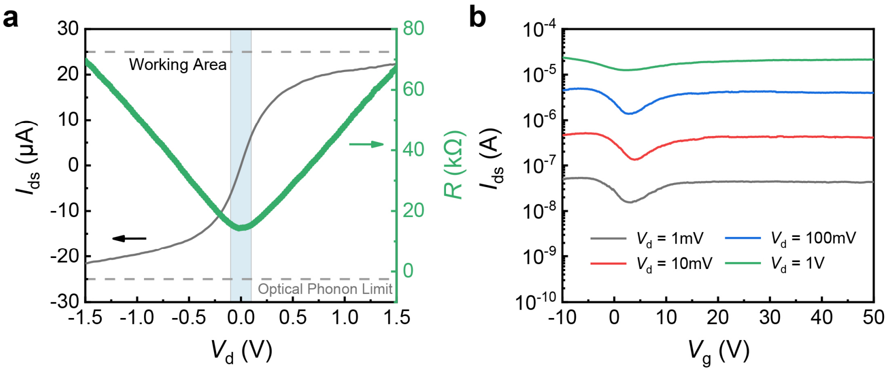

Supplementary Fig. 11 Current carrying capacity of the semimetal SWCNT. a, The I-V and R-V curves of the metallic SWCNT electrode. The I_{ds} tends to be saturated with the increase of V_d owing to the optical phonon limit. The *R*-*V* relationship reveals that the resistance of CNT electrode is approximately constant under low voltage ($V_d < 100$ mV), in which case it can carry more than 5 μA current. **b**, The transfer characteristics of the metallic SWCNT electrode under different bias voltage.

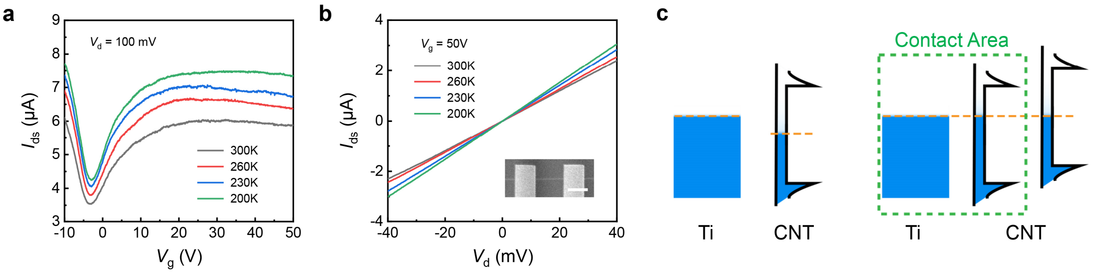

Supplementary Fig. 12 Temperature-dependent electrical transport properties of the semimetal SWCNT. The temperature-dependent transfer curves (**a**) and output curves (**b**) of the semimetal SWCNT. Inset is the SEM image of the CNT device. Scale bar: 2 μm. The conductivity of semimetal SWCNT increases with the temperature decreases, showing good metallicity and indicating that there is no potential barrier at the metal/CNT interface. **c**, Band diagrams of the metal/CNT junctions before and after contact formation.

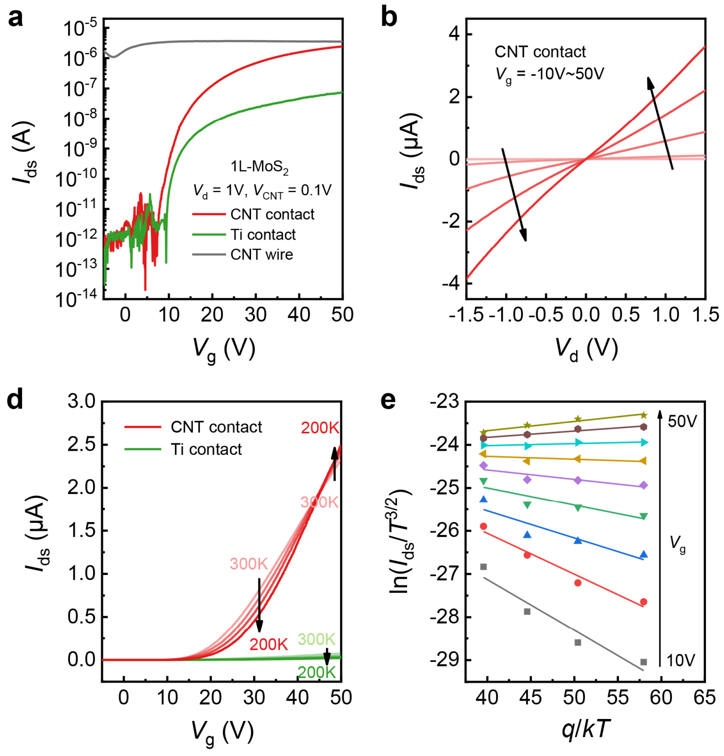

Supplementary Fig. 13 Electrical transport measurements of monolayer MoS_2 FETs with 1D semimetal contact. **a**, Room-temperature transfer characteristics of a monolayer MoS_2 FET with SWCNT (red) and Ti (green) contacts on 300nm SiO_2 dielectrics. **b**, Output characteristics of the MoS_2 FET with SWCNT contacts. **c**, Output characteristics of the MoS_2 FET with Ti contacts. **d**, Temperature-dependent transfer characteristics of the MoS_2 FET with SWCNT (red) and Ti (green) contacts. **e**, Arrhenius plots of the CNT-contacted monolayer MoS_2. **f**, Gate-dependent barrier height of the CNT/MoS_2 (red) and Ti/MoS_2 (green) interfaces.

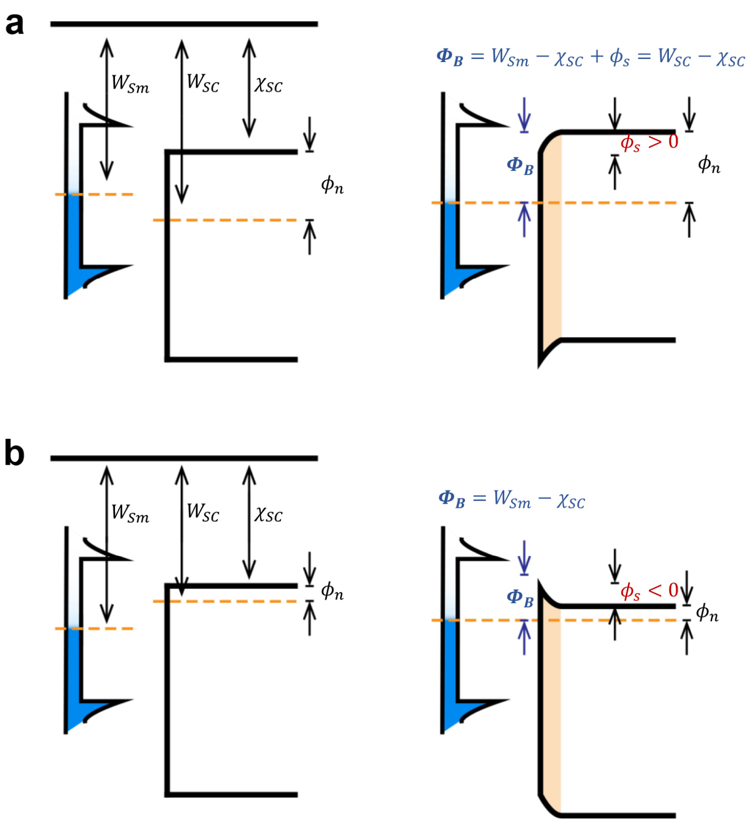

Supplementary Fig. 14 Band diagrams of the semimetal-semiconductor junctions for the surface potential of semiconductor $\phi_s > 0$ (**a**) and $\phi_s < 0$ (**b**).

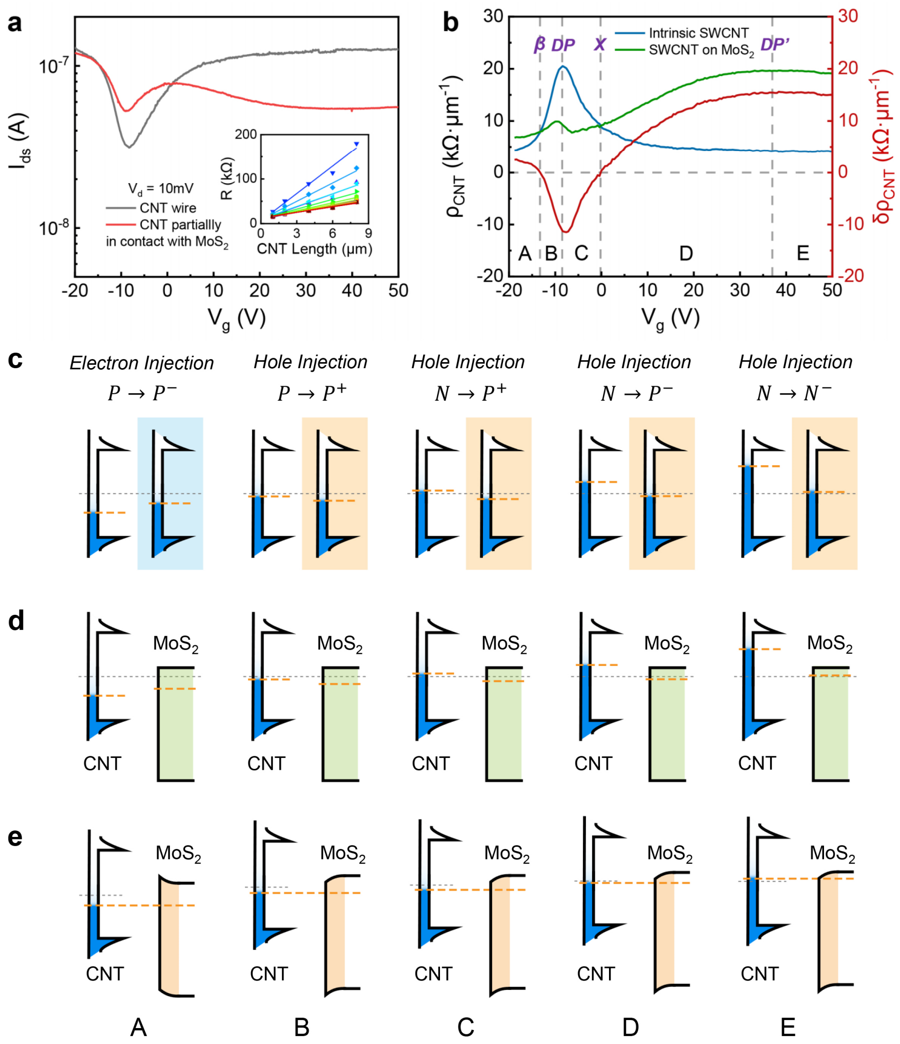

Supplementary Fig. 15 CIFS analysis of the CNT-MoS$_2$ junction. a, Transfer characteristics of the two SWCNT segments. Inset is the transfer length model plot of total resistance versus channel length corresponding to V_g from -20V to 50V. **b**, Extracted $\rho\&\delta\rho - V_g$ curves of the metallic SWCNT, all the data was measured under V_d = 100mV. **c**, Schematic of the contact-induced Fermi level shift behavior in metallic SWCNT under different V_g. Each pair of band diagrams represents the position change of Fermi level of CNT before and after contact with MoS$_2$. The orange and blue shaded background represents the contact-induced P-type (hole injection) and N-type (electron injection) doping, respectively. **d**, Schematic illustration of the deduced Fermi level positions of CNT and MoS$_2$ under different V_g before junction formation. **e**, Band diagrams of the CNT/MoS$_2$ heterostructure under different V_g after junction formation.

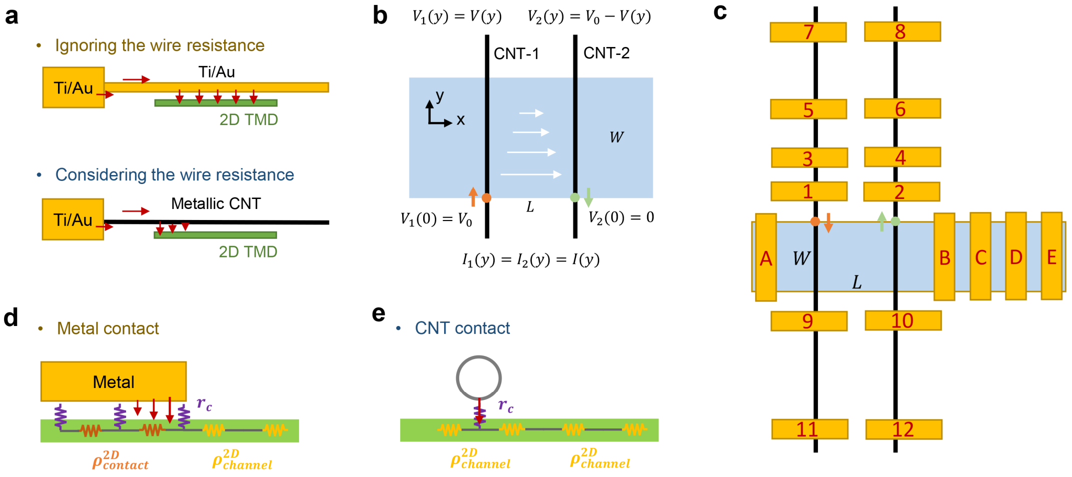

Supplementary Fig. 16 Model. a, Schematic of the current injection (side view). For metal Ti/Au, the wire resistance can be neglected and the current is injected into the channel evenly. For semimetal SWCNT, the wire resistance should be considered, and the current injected into the channel decreases gradually. **b**, Schematic of the simplified model and boundary conditions. **c**, Device design drawings. Schematic of the current injection (front view). **d**, Current crowding at the 3D/2D metal contact edge region. **e**, Current injected into channel at almost one point at the 1D-2D interface, which improves the injection efficiency.

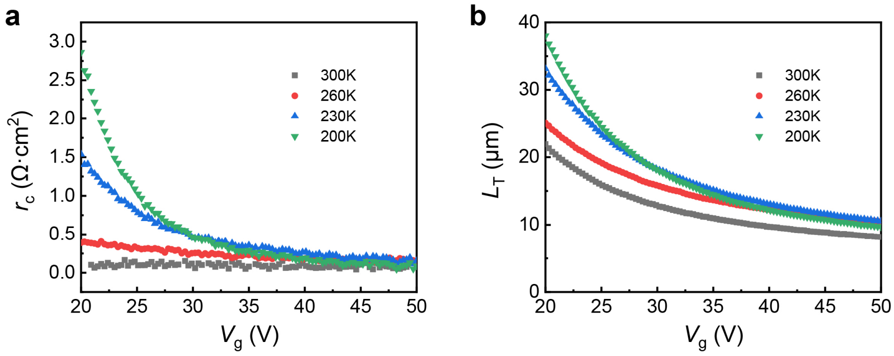

Supplementary Fig. 17 Temperature-dependent r_c and L_T. a, The 1D-2D interface contact resistivity r_c measured at different temperature. **b**, The calculated transfer length L_T at different temperature. According to the Boltzmann distribution, the tail of hot electrons decreases with decreasing temperature, which means that a larger positive gate potential is required for the hot electrons to cross the potential barrier. Therefore, the r_c increases with the decrease of temperature at V_g = 20V because of the Schottky barrier at the CNT/MoS_2 interface. And the same value of r_c can be achieves at V_g = 50V due to the formation of Ohmic contact.

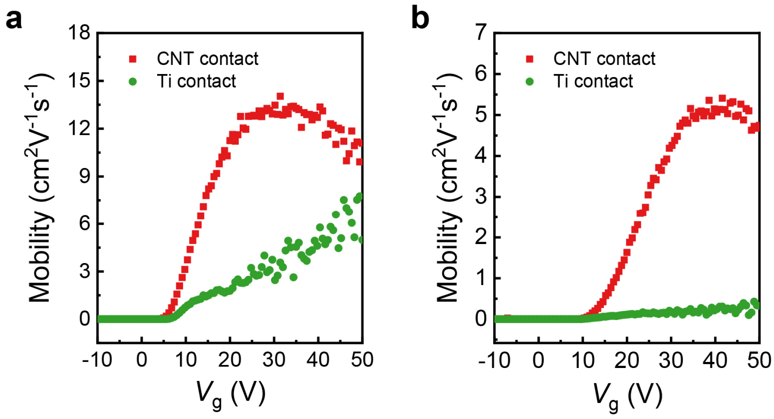

Supplementary Fig. 18 Mobility. The field effect mobility calculated using the equation $\mu = [dI_{ds}/dV_g] \times [L/WC_{ox}V_d]$ directly for the 4L-MoS_2 FET (**a**) and 1L-MoS_2 FET (**b**).

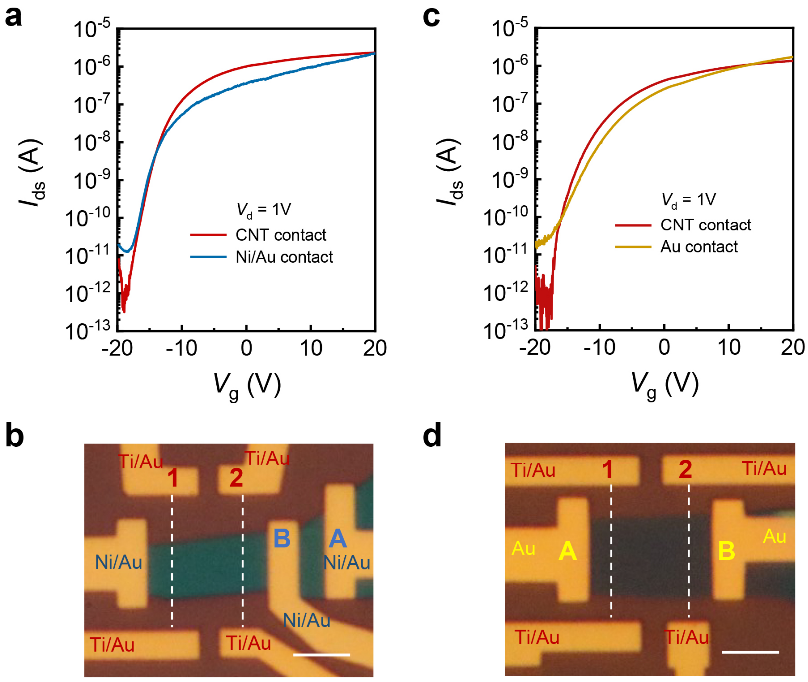

Supplementary Fig. 19 Comparison of the 1D semimetal contacts and Ni, Au contacts. Transfer characteristics (**a**) and optical image (**b**) of a MoS_2 FET with SWCNT (red, electrodes 1 and 2) and Ni (blue, electrodes A and B) contacts on P-type Si/300 nm SiO_2 substrate. Transfer characteristics (**c**) and optical image (**d**) of a MoS_2 FET with SWCNT (red, electrodes 1 and 2) and Au (yellow, electrodes A and B) contacts. Scale bar: 6 μm.

Supplementary References

1 Yu, L. *et al.* Graphene/MoS2 hybrid technology for large-scale two-dimensional electronics. *Nano letters* **14**, 3055-3063 (2014).

2 Franklin, A. D. & Chen, Z. Length scaling of carbon nanotube transistors. *Nature nanotechnology* **5**, 858-862 (2010).

3 Tsuneya Ando, T. N. Impurity Scattering in Carbon Nanotubes – Absence of Back Scattering –. *Journal of the Physical Society of Japan* **67**, 1704-1713 (1998).

4 Tsuneya Ando, T. N., and Riichiro Saito. Berry's Phase and Absence of Back Scattering in Carbon Nanotubes. *Journal of the Physical Society of Japan* **67**, 2857-2862 (1998).

5 Paul L. McEuen, M. B., David H. Cobden, Young-Gui Yoon, and Steven G. Louie. Disorder, Pseudospins, and Backscattering in Carbon Nanotubes. *Physical review letters* **83**, 5098-5101 (1999).

6 Li, X. *et al.* Gate-tunable contact-induced Fermi-level shift in semimetal. *PNAS* **119**, e2119016119, doi:doi:10.1073/pnas.2119016119 (2022).

7 Berger, H. H. Models for contacts to planar devices. *Solid-Stare Electronics* **15**, 145-158 (1972).

8 D.B. Scott, W.R. Hunter & Shichijo, H. A transmission line model for silicided diffusions: Impact on the performance of VLSI circuits. *IEEE J. Solid-State Circuits* **17**, 281-291 (1982).

9 Simmons, J. G. Generalized Formula for the Electric Tunnel Effect between Similar Electrodes Separated by a Thin Insulating Film. *Journal of Applied Physics* **34**, 1793-1803, doi:10.1063/1.1702682 (1963).

10 Simmons, J. G. Electric Tunnel Effect between Dissimilar Electrodes Separated by a Thin Insulating Film. *Journal of Applied Physics* **34**, 2581-2590, doi:10.1063/1.1729774 (1963).

11 Ilani, S., Donev, L. A. K., Kindermann, M. & McEuen, P. L. Measurement of the quantum capacitance of interacting electrons in carbon nanotubes. *Nature Physics* **2**, 687-691 (2006).

12 Parkash, V. & Goel, A. K. Quantum Capacitance Extraction for Carbon Nanotube Interconnects. *Nanoscale research letters* **5**, 1424-1430, doi:10.1007/s11671-010-9656-4 (2010).

13 Jorio, A. *et al.* Structural (n, m) determination of isolated single-wall carbon nanotubes by resonant Raman scattering. *Physical review letters* **86**, 1118-1121 (2001).
