# Supplementary material for: One-dimensional semimetal contacts to two-dimensional semiconductors
Source: Nat Commun. 2023 Jan 7;14:111. doi: 10.1038/s41467-022-35760-x (PMC9825564; doi:10.1038/s41467-022-35760-x)
Supplement: Supplementary file 3 — Description of Additional Supplementary Files [file 41467_2022_35760_MOESM3_ESM.pdf]

Description of Additional Supplementary Files

File Name: Supplementary Movie 1
Description: The Supplementary Movie 1 shows that the entire individual ultralong CNT on Si substrate can be efficiently tracked with the optical microscope.
